# Biosynthesis of Nature-Inspired Unnatural Cannabinoids

**DOI:** 10.3390/molecules26102914

**Published:** 2021-05-14

**Authors:** Kevin J. H. Lim, Yan Ping Lim, Yossa D. Hartono, Maybelle K. Go, Hao Fan, Wen Shan Yew

**Affiliations:** 1 Synthetic Biology for Clinical and Technological Innovation, National University of Singapore, 28 Medical Drive, Singapore 117456, Singapore; kevinljh@u.nus.edu (K.J.H.L.); bchlimy@nus.edu.sg (Y.P.L.); yossa.dwi.hartono@nus.edu.sg (Y.D.H.); bchmdkg@nus.edu.sg (M.K.G.); fanh@bii.a-star.edu.sg (H.F.); 2 Synthetic Biology Translational Research Programme, Yong Loo Lin School of Medicine, National University of Singapore, 14 Medical Drive, Singapore 117599, Singapore; 3 Department of Biochemistry, Yong Loo Lin School of Medicine, National University of Singapore, 8 Medical Drive, Singapore 117597, Singapore; 4 Bioinformatics Institute, A*STAR, 30 Biopolis Street, Matrix #07-01, Singapore 138671, Singapore

**Keywords:** *Cannabis sativa*, cannabinoids biosynthesis, metabolic engineering, synthetic enzymology, natural products, cannabinoid receptors, drug design

## Abstract

Natural products make up a large proportion of medicine available today. Cannabinoids from the plant *Cannabis sativa* is one unique class of meroterpenoids that have shown a wide range of bioactivities and recently seen significant developments in their status as therapeutic agents for various indications. Their complex chemical structures make it difficult to chemically synthesize them in efficient yields. Synthetic biology has presented a solution to this through metabolic engineering in heterologous hosts. Through genetic manipulation, rare phytocannabinoids that are produced in low yields in the plant can now be synthesized in larger quantities for therapeutic and commercial use. Additionally, an exciting avenue of exploring new chemical spaces is made available as novel derivatized compounds can be produced and investigated for their bioactivities. In this review, we summarized the biosynthetic pathways of phytocannabinoids and synthetic biology efforts in producing them in heterologous hosts. Detailed mechanistic insights are discussed in each part of the pathway in order to explore strategies for creating novel cannabinoids. Lastly, we discussed studies conducted on biological targets such as CB1, CB2 and orphan receptors along with their affinities to these cannabinoid ligands with a view to inform upstream diversification efforts.

## 1. *Cannabis sativa* and Cannabinoids—An Introduction

Natural products and their derivatives make up more than half of all medicine we use today [1]. The organisms that make them range widely from plants to microorganisms such as bacteria and fungi. Their structural complexity and diversity often provide a wide range of bioactivities that can be harnessed as therapeutic agents for various diseases and indications. Cannabinoids are one such class of natural products originally isolated from the plant *Cannabis sativa* that offers a wide range of bioactivities such as anti-bacterial [2], anti-epileptic [3] and anti-tumor effects [4].

These chemical compounds are meroterpenoids characterized by a resorcinyl-polyketide core attached to a 10-carbon monoterpene [5]. They are found to interact with G protein-coupled receptors (GPCRs) such as cannabinoid receptor type 1 (CB1) receptors which are mainly found in the central nervous system (CNS), and cannabinoid receptor type 2 (CB2) receptors found predominantly in the peripheral nervous system (PNS). However, studies have also uncovered these receptors to be expressed in different tissues such as the adrenal gland, lungs, heart, spleen and tonsils [6]. The widespread expression of these receptors suggests that cannabinoids have the potential to target a wide range of biological targets in the human body. The class of cannabinoids derived from *C. sativa* was later termed phytocannabinoids due to the discovery of the endocannabinoids in humans that are also found to interact with the same GPCRs. Additionally, chemists also synthesized synthetic analogues of these compounds with interactions towards the same receptors and are hence termed the synthetic cannabinoids. This review will focus on the class of phytocannabinoids.

Although extracts of the *Cannabis* plant have been the subject of illicit use in the last few decades due to some psychoactive components such as ∆^9^-tetrahydrocannabinol (THC), it was first used as a traditional medicine and this suggests that there is therapeutic potential in this class of natural products. In the context of *Cannabis* extract, the “entourage effect” is often discussed. The term was first proposed in 1998, where it was observed that other metabolites and similar molecules increased the activity of the primary endogenous cannabinoids [7], but now more commonly refers to the synergistic enhancement of main cannabinoids from other components in the plant extract. However, there are differing views on whether this claim of polypharmacy has strong supporting evidence. Further discussion can be found in recent reviews and references therein [8,9,10,11].

Currently, *C. sativa*-derived cannabinoids are used clinically for spasticity treatment in multiple sclerosis patients who are non-responsive to other medications (Sativex^®^) in some European countries [12]. Most recently, Epidiolex^®^ became the first *Cannabis*-based drug to be approved by the US Food and Drug Administration (FDA) to reduce seizures in epilepsy patients with Dravet syndrome and Lennox–Gastaut syndrome [13]. These medicines are largely based on the two major cannabinoids produced by *C. sativa*, THC and cannabidiol (CBD), which are produced as the acidic forms tetrahydrocannabinolic acid (THCA) and cannabidiolic acid (CBDA), respectively, in the plant and readily decarboxylated during storage and heating [14]. These compounds represent only less than 2% of the total repertoire of naturally occurring phytocannabinoids available. To date, more than 100 cannabinoids have been identified from *C. sativa* alone [15], but research on the potential therapeutic uses of these other lesser known cannabinoids is still in its infancy. This is largely due to the low yield available to extract from the plant, and the inability to isolate a strain of *C. sativa* that selectively produces more of these rarer cannabinoids. In addition, different cultivars of *Cannabis* contain different proportions of cannabinoids. In the US, *Cannabis* is legally classified into hemp, or fiber-type (less than 0.3% THC) and drug-type (more than 0.3% THC).

Currently, cannabinoids for research and clinical use are mainly sourced from *C. sativa* cultivar extracts. This method of production requires significant resources and is an unsustainable avenue for harvesting cannabinoids, given its recent rapid expansion in demand worldwide. *Cannabis* farming requires extensive resources in order to cultivate these high-value plants in highly controlled environmental conditions. Research conducted by the Energy and Resources group in the Lawrence Berkeley National Laboratory in 2012 estimated that the cost of energy consumption from the indoor practice of *Cannabis* cultivation in the US alone amounted to around USD 6 billion annually. In addition, the carbon dioxide gas emission from this practice to produce an average kilogram of the final product was equivalent to that of 3 million cars [16]. Additionally, *Cannabis* farming contributes to the problem of illicit drug use due to the lure of recreational marijuana. According to a report by the American Civil Liberties Union, the US spent USD 3.6 billion annually in 2010 in *Cannabis* law enforcement [17].

These complications fuel the need to find an alternative method of production that is cheaper, and more environmentally, economically and socially sustainable. Synthetic biology has provided the world with a solution in producing specific chemical compounds at a lower cost and higher selectivity using engineered microbes. The success of synthetic biology companies such as Amyris and Antheia in producing traditionally difficult and expensive medicine such as artemisinin for the treatment of malaria, and opioids for a variety of disease indications, have laid the foundations for many other biotech firms to follow. Several *Cannabis*-cultivating companies have shored up collaborations with synthetic biologists with the same goal in mind, producing specific cannabinoids without the use of *C. sativa* cultivation. In recent years, several groups have published studies on the biosynthesis of cannabinoid precursors in micro-organisms such as bacteria and yeast strains [18,19]. In 2019, a group led by Keasling published a landmark paper on the total biosynthesis of the cannabinoids, THCA and CBDA, using simple sugars in yeast [20].

The establishment of the cannabinoids biosynthetic pathway in heterologous systems has created new opportunities for producing rare cannabinoids such as cannabinol (CBN) and cannabicyclol (CBL) for research into their therapeutic potential. Additionally, this has also accelerated the channel of uncovering novel cannabinoid-like analogues derivatized from these primary structures by reviewing the available chemical space. Cannabinoid analogues such as the ones produced by Luo et al. with different side chains could elicit novel responses in the human body and uncover unique therapeutic applications. This review aims to summarize the recent advances in the field of synthetic cannabinoid biology and subsequently examine further strategies for creating novel cannabinoids. Lastly, understanding how classical phytocannabinoids interact with downstream signaling targets such as CB1 and CB2 will inform upstream diversification efforts.

### Cannabinoid Biosynthetic Pathway

**Figure 1:**
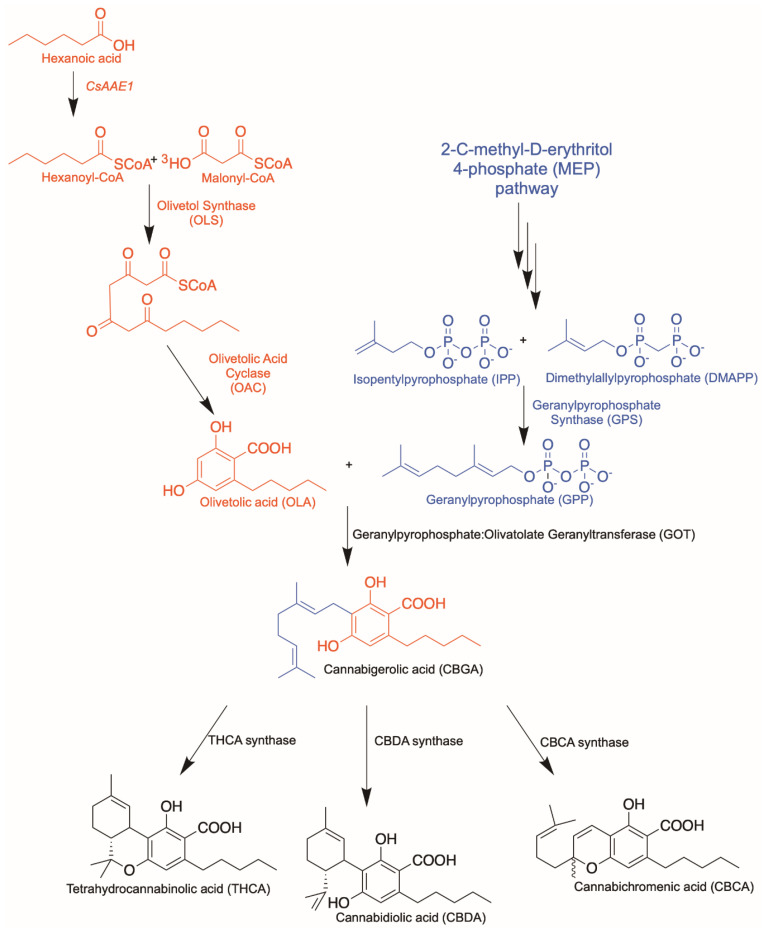
Cannabinoid biosynthetic pathway. Polyketide pathway is highlighted in red; isoprenoid pathway is highlighted in blue.

In order to engineer the biosynthesis of novel cannabinoids, it is vital to first understand the cannabinoid biosynthetic pathway (Figure 1). In *C. sativa*, hexanoyl-CoA is produced by an acyl-activating enzyme, *Cs*AAE1, from hexanoic acid derived through the fatty acid biosynthesis pathway [21]. Thereafter, a type III polyketide synthase named olivetol synthase (OLS) elongates one unit of hexanoyl-CoA using three units of malonyl-CoA in a manner characteristic of other PKSs [22]. However, unlike other polyketide synthases of its kind, OLS does not have a cyclization pocket and hence lacks the ability to cyclize the intermediate, a tetraketide-CoA thioester [23]. Instead, a cyclase enzyme, olivetolic acid cyclase (OAC), catalyzes the necessary C2 to C7 aldol condensation reaction to produce olivetolic acid (OLA) [24].

Next, a putative prenyltransferase enzyme transfers the C10 chain from geranyl pyrophosphate (GPP) to C3 of OLA to produce the first cannabinoid, cannabigerolic acid (CBGA). The native prenyltransferase from *C. sativa* that completes the step was identified as *Cs*PT1 or geranylpyrophosphate: olivetolate geranyltransferase (GOT) in 1998 [25]. However, this protein is predicted to be a transmembrane protein with a plastid targeting signal due to its plantal origin. Several groups have attempted heterologous expression of a functional *Cs*PT1 without much success. Luo et al. found a functionally active prenyltransferase, named *Cs*PT4, from *C. sativa* that produces the CBGA from GPP and OLA [20]. GPP is presumably supplied through the 2-C-methyl-D-erythritol 4-phosphate (MEP) pathway in *C. sativa* due to the plastid localization tag found on the sequences of subsequent prenyltransferase and cannabinoid synthases.

Finally, CBGA serves as a branch-point in the pathway where several cannabinoids such as THCA, CBDA or cannabichromenic acid (CBCA) can be produced. This is achieved through differential cyclization of the C10 moiety from GPP via the actions of independent synthases. Categorically, the biosynthetic pathway can be dissected into three distinct functional parts: the polyketide pathway producing OLA, the isoprenoid pathway together with the prenyltransferase step producing CBGA as the first cannabinoid, and the last cyclization step producing various cannabinoids from CBGA. Discussions on synthetic biology approaches will be organized according to these individual enzymatic steps in the pathway, along with structural and mechanistic insights, to facilitate the subsequent framing of strategies for producing novel cannabinoids.

## 2. Olivetolic Acid Derivatives

The amino acid sequence of the type III polyketide synthase, OLS, from *C. sativa* was identified in 2009. It was noted then, that OLS does not produce OLA, and only forms three reaction products: a triketide pyrone, a tetraketide pyrone and olivetol, the decarboxylated alcohol form of OLA [22]. It was understood at that time that cannabinoid synthases producing THCA and CBDA produced downstream in the pathway from CBGA could not catalyze the reaction using the alcoholic form, CBG [26,27]. Hence, this would mean that there is a gap in the pathway that produces the corresponding acidic polyketide upstream, OLA. The missing link in the upstream polyketide pathway was only uncovered with the identification of OAC in 2012 by Gagne et al. [24]. OAC was the first polyketide cyclase to be found in plants. It utilizes the putative tetraketide-CoA product from OLS as a substrate and cyclizes it to form OLA. This interplay between type III polyketide synthases and cyclase enzymes to produce structural diversity in natural products was an exciting development in the established field of polyketide synthases.

**Figure 2:**
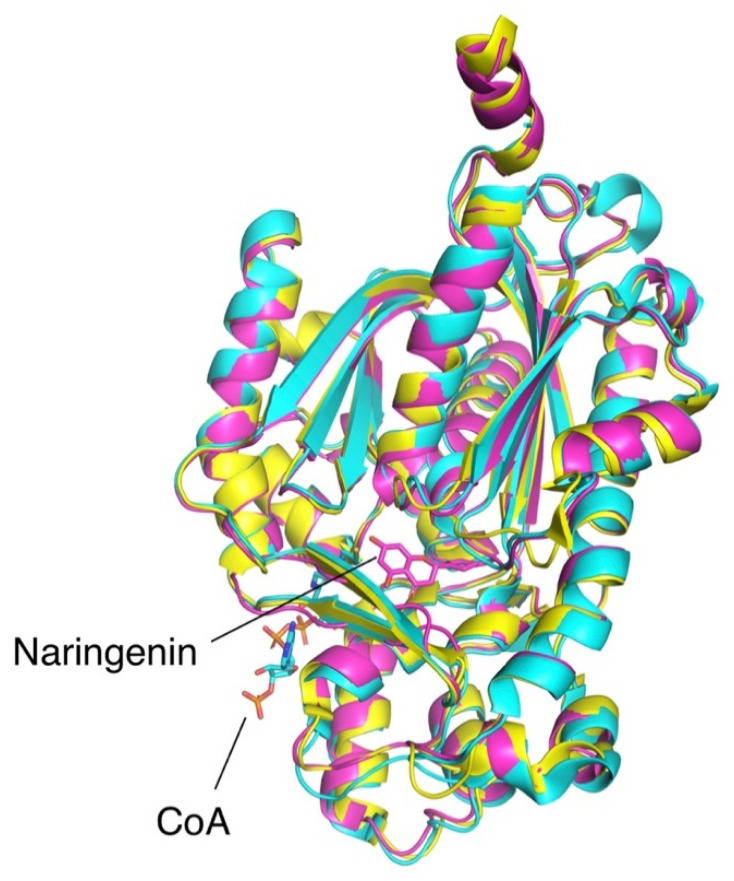
Superimposed structures of OLS (cyan, PDB 6GW3 [23]), stilbene synthase (yellow, PDB 1U0U [28]), and chalcone synthase (purple, PDB 1CGK [29]). Shown ligands CoA and naringenin are co-crystallized with OLS and chalcone synthase, respectively.

The structure of OLS was finally resolved more than a decade after its initial characterization [23]. Type III PKSs have highly similar structural features, as observed in the well-characterized stilbene synthase [28] and chalcone synthase [29] (Figure 2). Both PKS mentioned along with OLS catalyze highly similar reactions and form a tetraketide-CoA thioester intermediate from three rounds of chain-elongating condensations. However, the reaction mechanism seems to diverge after the formation of the elongated tetraketide intermediate. Chalcone synthase (CHS) adopts a C6 to C1 Claisen condensation to form naringenin, a chalcone product, while OLS and stilbene synthase seems to adopt a spontaneous C2 to C7 decarboxylative aldol condensation to form the stilbene products, olivetol and resveratrol, respectively.

**Figure 3:**
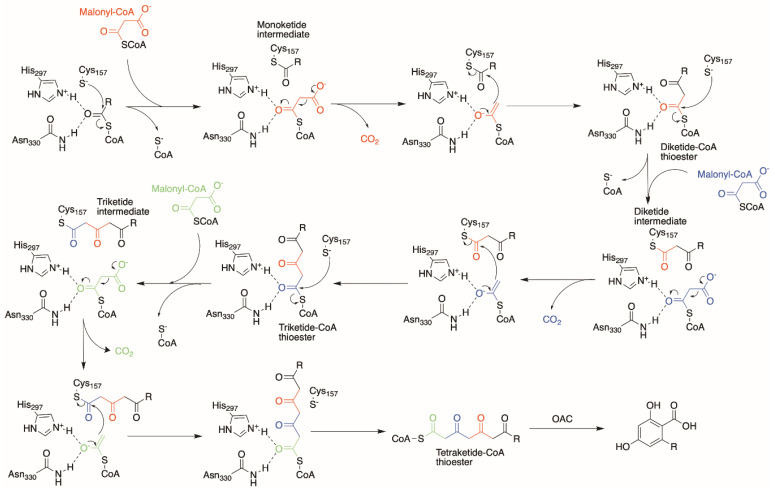
Proposed OLS mechanism forming a tetraketide-CoA thioester product for OAC to cyclize into a resorcinolic ring such as OLA.

The active site of OLS is bi-lobed, with each lobe accommodating the starter-molecule binding and the elongating polyketide chain. Using analogous information from previous type III PKSs [28,29,30], we proposed the following reaction mechanism for OLS involving the Cys157/His297/Asn330 catalytic triad (Figure 3). Upon binding of the starter-CoA unit at the active site, Cys157 activated by His297 attacks the thioester linkage between the starter unit and the CoA-moiety, forming a monoketide intermediate attached to Cys157. The first malonyl-CoA extender unit then diffuses into the active site and is decarboxylated to form an acetyl-CoA carbanion. The nucleophilic acetyl-CoA carbanion then attacks the thioester linkage between the starter unit and Cys157, thereby forming a diketide-CoA intermediate. Subsequently, Cys157 recaptures the elongated diketide unit and releases the CoA-moiety to reinitialize the process for two more rounds of elongation (Figure 3).

However, unlike its orthologues, OLS lacks a specific cyclization mechanism for the elongated tetraketide-CoA product and releases it from its active site. The three reaction products found from OLS activity are postulated to be the result of a spontaneous C5-oxygen to C1 lactonization event that occurs in the triketide-CoA intermediate from two rounds of elongation to form the triketide pyrone, and differential spontaneous cyclization to form tetraketide pyrone and olivetol from a tetraketide-CoA product [22]. It was found later that OAC then utilizes this tetraketide-CoA product from OLS for a C2–C7 aldol cyclization without decarboxylation to produce OLA, as previously mentioned [24].

**Figure 4:**
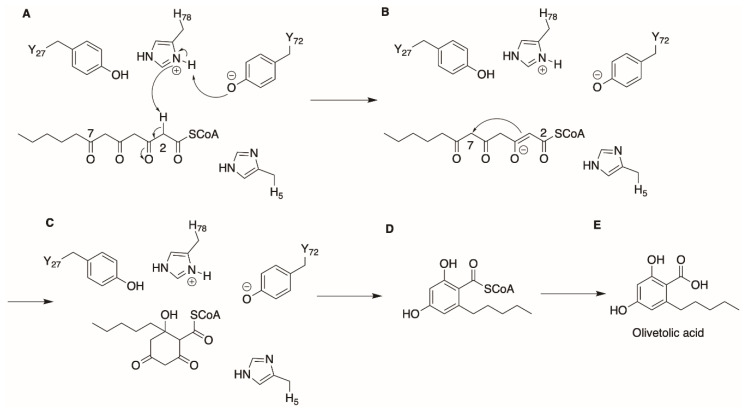
Proposed OAC cyclization mechanism to form OLA. (**A**) Extraction of proton by His78 from C2 of substrate. (**B**,**C**) C2 to C7 aldol condensation facilitated by reabsorption of proton from His78 by carbonyl O on C7. (**D**,**E**) Aromatization, release of CoA thioester and subsequent release of OLA product.

OAC was the first polyketide cyclase observed in plants. Several polyketide cyclases had previously been uncovered in bacteria, such as SnoaL from *Streptomyces nogalater* [31,32], the tetracenomycin aromatase/cyclase (Tcm ARO/CYC) [33,34] and the tetracenomycin F2 cyclase (TcmI) [35,36] from *Streptomyces glaucescens*. These enzymes catalyze aldol condensation reactions, causing the aromatization of their substrates. A closer look at the structure of OAC reveals that it is structurally similar to bacterial polyketide cyclases from *Streptomyces* sp., belonging to the dimeric α + β barrel (DABB) protein family [37]. It was reported that OAC does not interact physically with OLS for its catalytic action, and it accepts the linear 3, 5, 7-trioxododecanoyl-CoA product from OLS as substrate [22]. By solving the X-ray crystal structure, and with insights obtained through a series of site-directed mutagenesis experiments, Yang et al. proposed a reaction mechanism for OAC that uses an acid/base catalyst reaction to cyclize its substrate, as depicted in Figure 4 [37]. Facilitated by Tyr72, His78 first conducts a nucleophilic attack on the C2 of the tetraketide-CoA substrate to produce an enolate intermediate, as seen in Figure 4B. Next, the unstable enolate intermediate then attacks its C7 carbonyl carbon and a proton abstraction from His78 releasing water constitutes the aldol condensation reaction and cyclization. Subsequently, release of the CoA-linked intermediate from OAC followed by the spontaneous aromatization and cleavage of the CoA bond forms its product OLA.

A few groups have attempted the heterologous biosynthesis of OLA and its analogues since the identification of OLS and OAC. In 2018, Tan et al. published an article describing the synthetic production of OLA in *Escherichia coli* from glycerol [18]. Using a β-oxidation reversal pathway and the acetyl-CoA carboxylase (ACC) for hexanoate and malonyl-CoA precursor supply, coupled with OLS and OAC enzymes, they were able to produce up to ~80 mg/L of OLA in vivo using optimized fermentation conditions. Gülck et al. attempted OLA biosynthesis using the tobacco plant *Nicotiana benthamiana* by heterologous expression of OLS and OAC and observed the production of OLA and a glucosylated analogue, olivetolic acid glucoside. They postulated that this derivative occurs through the actions of uridine diphosphate glucosyltransferases (UDP-UGT), present in *N. benthamiana*, that are able to act on the OLA product as a substrate [38]. The glucosylation modification on OLA and its downstream cannabinoids may increase the aqueous solubility of these hydrophobic compounds and hence improve their bioavailability as medicines.

With the biosynthetic pathway for OLA elucidated and well-characterized, efforts to produce novel cannabinoids downstream using novel cannabinoid precursors such as OLA analogues can be attempted. In order to serve as cannabinoid precursors, analogues of OLA will need to retain a few key features for the binding and activation of downstream CB receptors. The dihydroxystilbene acid feature with hydroxyl groups at C2 and C4, accorded by the use of malonyl-CoA as extenders, and a carboxylic acid attached to the resorcinolic ring, is key for further downstream processing of prenylation and oxidative cyclization. The following sections discuss different strategies for producing OLA analogues with the intent of producing novel cannabinoids.

### 2.1. Precursor-Directed Combinatorial Biosynthesis

Type III PKS enzymes have been shown to be highly promiscuous. Lim et al. showed that the stilbene synthase mutant, 18xCHS, was able to utilize acyl-CoA esters derived from butanoic acid, hexanoic acid, heptanoic acid, and even 3-hexenoic acid as starter units to form novel polyketides using malonyl-CoA as extenders, although the identities of the polyketides were not characterized [39]. The type III PKS from rice *Oryza sativa* (OsPKS) was screened against 70 starter and 12 extender acyl-CoA thioesters which yield a total of 840 different combinations, of which 315 combinations were found to produce a new potential polyketide (38% of total matrix) [30]. The authors attributed this promiscuity profile to substrates with a degree of “likeness” to its native substrates, 4-hydroxycinnamyl-CoA and malonyl-CoA. Out of the 315 combinations, the authors characterized two polyketides, bisnoryangonin and 26OH, which were shown to exhibit antimicrobial activities. Taura et al. characterized OLS from *C. sativa* to be able to accept four different substrates as its starter unit, the native hexanoyl-CoA, butyryl-CoA, isovaleryl-CoA, and octanoyl-CoA, as they were able to observe a triketide pyrone product from using these starter units [22]. However, a resorcinol product was observed only from using hexanoyl-CoA and butyryl-CoA as starter units, producing olivetol and divarinol. This meant that when isovaleryl-CoA and octanoyl-CoA were used as the starter units, its reaction catalysis ended with only two rounds of extension, producing only the corresponding triketide pyrones of these substrates. However, there have been no reports to date of attempts at a more exhaustive screen of OLS with a larger library of acyl-CoA thioesters as starter or extender substrates. Understandably, the process in making OLA in nature requires the synergistic action of both OLS and the cyclase enzyme, OAC. Screening OLS against a library of potential substrates also requires a compatible cyclase enzyme such as OAC to cyclize the corresponding tetraketide intermediate via a C2–C7 aldol condensation; is also required to produce corresponding analogues of OLA. The task at hand is limited not just by the substrate promiscuity of OLS, but also OAC. However, such work is important in elucidating the structural aspects of the substrate selectivity of OLS. This will, in turn, pave the way for novel cannabinoids to be produced through a library of OLA analogues with different side chains on C6 of the aromatic ring.

### 2.2. Protein Engineering

Exploring the substrate selectivity profile of OLS allows us to understand the extent of “how promiscuous” the enzyme can be in accepting different substrates. However, there is a limit in an enzyme’s substrate promiscuity profile that is governed by steric and polarity constraints. Protein engineering allows us to push the boundaries of an enzyme’s innate substrate specificity by introducing favorable mutations that allows the enzyme in context to accept a different substrate that it was previously unable to accept, or even to alter its reaction mechanism. Traditional methods of protein engineering, such as directed evolution, require a selection method, such as a toxicity or biomarker screen, to facilitate the easy selection of clones with mutant proteins that carry the desired trait. However, it may not be easy to design a robust selection method for picking out mutants that produce novel products with unknown toxicity or pharmacological profiles. In such instances, a rational structure-based approach in protein engineering would be useful.

As previously mentioned, close orthologues of OLS, chalcone synthase and stilbene synthase, catalyze similar reactions except for the difference in starter unit preference and cyclization mechanism. Structural alignment of these orthologues with OLS allows us to identify the non-conserved residues between the orthologues and may explain the differences in substrate specificity and cyclization patterns. Unfortunately, Kearsey et al. was unable to identify the residues responsible for the differences in cyclization pattern among the orthologues when they performed targeted single mutational changes of the non-conserved residues in OLS to the corresponding residues in chalcone synthase [23]. Given the highly complex nature of the reaction catalyzed, it is likely that multiple residues were responsible for the catalytic differences in cyclization instead of single key residues. This is evident in the protein engineering project undertaken by Austin et al. where 18 amino acid residues were mutated in order to convert an alfalfa CHS to a corresponding stilbene synthase catalyzing a C2–C7 decarboxylative aldol condensation [28]. The “aldol switch” identified, 18 amino acid residues along with a network of hydrogen-bonding configuration, was responsible for converting the chalcone-predominant product to a stilbene-predominant product. Future work investigating a more thorough set of the amino acid residue differences among these orthologues would be useful for future protein engineering work in creating novel OLA analogues.

An interesting strategy for protein engineering of PKS enzymes governing substrate selectivity and catalytic activity is domain swapping. Domain swapping is the substitution of a domain in a monomeric protein with another domain of an identical or similar protein. Since the first instance of domain swapping was reported in diphtheria toxin dimeric form, 3D domain swapping as a protein engineering tool has been used to modify an enzyme’s bioactivity function such as substrate specificity and catalytic activity [40]. Liu and Eisenberg provided an excellent review on the current knowledge and possibilities of 3D domain swapping [41]. The term domain can be redefined as a functional unit of specific catalytic activity. In the context of PKS protein engineering, this is evidenced in many protein engineering studies of model PKSs such as the 6-deoxyerythronolide synthase (DEBS) where the swapping of domains within the enzyme produces varied products [42]. Future studies on OLS structure–function relationships will facilitate the engineering of catalytic domains into the enzyme such as introducing thioesterase activity in OLS to perform the C2–C7 aldol condensation reactions of the tetraketide product and factors governing substrate selectivity.

### 2.3. Orthologues of Interest

Enzyme orthologues may behave differently when exposed to the same set of substrates. This was evidently observed in chalcone and stilbene synthases, which are both able to accept coumaroyl-CoA and malonyl-CoA as the starter and extender unit, yet diverge to produce naringenin and resveratrol, respectively. This similar principle could be applied to screening efforts of a library of different OLS orthologues for the production of novel OLA analogues with a common resorcinyl core that may serve as substrates for the following steps in the cannabinoid biosynthetic pathway.

An example of such an orthologue is the type III PKS from *Rhododendron dauricum*, orcinol synthase (ORS), which catalyzes a similar reaction to OLS except that it uses acetyl-CoA instead of hexanoyl-CoA for its starter unit [43]. Interestingly, ORS alone also produces the decarboxylated resorcinol equivalent, orcinol as its major product, much like OLS producing olivetol as the major product. When OAC from *C. sativa* was added in an in vitro reaction with ORS, the orsellinic acid (OSA) production increased significantly, which suggests that an unidentified polyketide cyclase much like OAC could be present in *R. dauricum*. It would be interesting to study amino acid differences between the cyclase from *R. dauricum* and OAC from *C. sativa* and elucidate how that contributes to substrate specificity. This phenomenon displays the close resemblance between the two enzymes separated by a speciation event that divides them into different species.

Hydrangic acid and prelunularic acid are polyketides thought to be produced by putative stilbene synthases that utilizes coumaroyl-CoA and dihydrocoumaroyl-CoA, respectively, as the starter units, and three units of malonyl-CoA as the common extender unit. A unique property of these reactions is the ability of the putative type III PKS to catalyze C2–C7 aldol condensation with retention of the carboxylate functional group to form hydrangic acid and prelunularic acid. This is presumed to be attributed to either the synergistic action of unidentified polyketide cyclases in *Hydrangea* or to reduction via a ketoreductase (KR) domain in the enzyme due to the lack of the hydroxyl group at the C5 carbonyl [5,44]. The ability of the PKS to perform a non-decarboxylative aldol condensation after chain elongation is important in cannabinoid biosynthesis, as the carboxylate functional group is vital in interacting with cannabinoid synthases later in the biosynthetic pathway [45]. Orthologues that may perform the rare non-decarboxylative aldol condensation step will circumvent the need for an additional cyclase step, improving yields in a heterologous metabolic pathway by reducing the extent of loss of intermediates.

**Figure 5:**
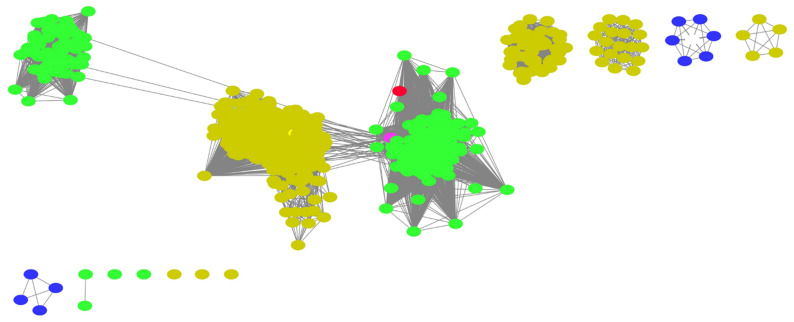
SSN generated using OAC amino acid sequence as a template with 40% sequence identity using EFI-EST webtool [46]. Green nodes represent sequences from plant species, yellow from bacterial species and dark purple from other *Eukaryota* species. OAC reference sequence is colored red. Uncharacterized sequence from *M. polymorpha* is colored magenta.

As mentioned previously, a replacement of OLS in the pathway producing a different product may require the synergistic action of a corresponding polyketide cyclase, such as OAC, to perform the C2–C7 non-decarboxylative aldol condensation to form the resorcinolic acid scaffold for further downstream catalysis. The aforementioned biosynthesis of orsellinic acid by the action of ORS in *R. dauricum* still lacks clarity on an unidentified cyclase enzyme. Polyketide cyclases are relatively new to the world of polyketide biosynthesis and until more orthologous sequences are identified, this will remain a bottleneck in finding replacements for the PKS in the cannabinoid biosynthetic pathway. Webtools such as the Enzyme Similarity Tool from the Enzyme Function Initiative (EFI-EST) could aid in identifying orthologues of polyketide cyclases such as OAC that are not well-characterized or annotated. The web-tool uses databases such as UniProt to identify amino acid sequences related to the query sequence in terms of function and reaction catalysis. It generates a sequence similarity network (SSN) which allows for the easy visualization of the relationship between sequences across different species [46]. The SSN generated for OAC, as shown in Figure 5, is relatively small, with only 581 sequences identified, which is unsurprising given their recent discovery. Another observation is that the majority of these orthologous sequences are found in plants (green nodes) and bacteria (yellow nodes) species. The presence of known and characterized orthologous sequences such as At5g22580 from *Arabidopsis thaliana* [47] and SP1 from *Populus tremula* [48], as well as uncharacterized sequences from liverwort species such as *Marchantia polymorpha* (colored magenta in Figure 5), which is also known to produce the stilbene acids, prelunularic acid and lunularic acid [5], lends credibility and demonstrates utility to the approach. Through this, sequences from diverse origins with different substrate specificities could be uncovered that may serve as replacements in the biosynthetic pathway.

## 3. Aromatic Prenyltransferases

“Prenylation” is the enzymatic transfer of the hydrophobic side chain from a terpene diphosphate to another biomolecule [49]. Prenyl groups can be small, five-carbon units (C5) such as the dimethylallyl or isopentenyl moieties, or larger components with multiple five-carbon units such as the geranyl (C10) or farnesyl (C15) groups. Although a lot of research has been carried out on protein prenyltransferases and the significance of this post-translational modification step in cellular signaling activities [50], not much is known about small aromatic molecule prenyltransferases. Aromatic prenylation plays a critical role in the biosynthesis of diverse bioactive secondary metabolites in plants, fungi and bacteria, with the prenyl group enhancing the uptake and biological activities of the aromatic core residue [51]. CBGA is the first cannabinoid produced in the cannabinoid biosynthetic pathway in *C. sativa*, and is formed by a C–C Friedel–Craft alkylation of OLA at the C3 position [52].

CBGA, the first branch-point in the cannabinoid biosynthetic pathway, was found to be produced from OLA and GPP by GOT in the *Cannabis* plant more than two decades ago [25], and a *Cannabis* GOT (*Cs*PT1) was patented 16 years later [53]. In the patent, *Cs*PT1 was cloned and expressed in *Spodoptera frugiperda* 9 insect cells, and was able to produce CBGA and the side product 5-geranyl olivetolate when insect cell microsomes containing *Cs*PT1 were incubated with GPP, OLA and MgCl_2_ [53]. Nevertheless, several groups tried to express and characterize a functional *Cs*PT1 in other hosts but were unsuccessful [20,38]. By analyzing published *Cannabis* transcriptomes, Luo et al. found a functionally active prenyltransferase, named *Cs*PT4, from *C. sativa*, that biosynthesizes CBGA from GPP and OLA. Interestingly, *Cs*PT4 only shared 62% homology to *Cs*PT1, and is predicted to have eight transmembrane helices [20]. *Cs*PT4 was also found to be substrate-promiscuous for its aromatic prenyl-acceptor as it could accept OLA analogues derived from butanoic acid, pentanoic acid, 5-hexenoic acid, heptanoic acid, 4-methylhexanoic acid, and 6-heptynoic acid, while using GPP as the prenyl-donor producing CBGA analogues with varying side chains at the C6 position. Although the heterologously expressed *Cs*PT4 can localize to the purified microsomal fraction from yeast and facilitate the determination of enzyme kinetics when the microsomal preparation was incubated with OLA and GPP, the lack of a crystal structure hinders efforts to gain mechanistic insights into its substrate selectivity. Concurrently, research by another group showed that *Agrobacterium tumefaciens* mediated the transient expression of *Cs*PT4 in *N. benthamiana*, which resulted in CBGA production when GPP and OLA were delivered as substrates by leaf infiltration [38]. The gene loci of *Cs*PT1 and *Cs*PT4 are in close proximity, and both are highly and selectively expressed in *Cannabis* trichomes where cannabinoids accumulate, suggesting that *Cs*PT1 and *Cs*PT4 might be functionally redundant in *C. sativa*.

### 3.1. Plant Aromatic Prenyltransferases

Since the first plant prenyltransferase was characterized and expressed by Yazaki et al. in 2002, only about 67 plant aromatic prenyltransferases have been identified and functionally characterized [54] in the past decade. Plant aromatic prenyltransferases often show remarkable substrate specificity, catalyzing the regio- and stereospecific prenylation of aromatic substrates. For instance, the prenyltransferase from *Lotus japonicus* is dimethylallyl diphosphate (DMAPP) and genistein-specific and solely produces 6-prenylgenistein, which has anti-fungal properties [55]. Most of the known plant aromatic prenyltransferases are highly specific to DMAPP as its prenyl donor, although there are a few exceptions. Apart from *Cs*PT1 and *Cs*PT4 from *C. sativa* [20,53], HlPT1L and HlPT2 involved in bitter acid biosynthesis in *Humulus lupulus* (hops; a close relative of *Cannabis*), p-hydroxybenzoate geranyltransferases (LePGT-1 and LePGT-2) involved in shikonin biosynthesis in purple gromwell (*Lithospermum erythrorhizon*) [56], were all found to be able to accept GPP as the prenyl donor to transfer the geranyl moiety to a range of aromatic substrates.

The first plant farnesyltransferase, *Rd*PT1, was discovered in *R. dauricum*, which produces grifolic acid (GFA) from farnesyl pyrophosphate (FPP) and orsellinic acid. The farnesyl moiety of GFA can undergo oxidative cyclization to form daurichromenic acid, which has potent anti-HIV and anti-inflammatory activities [57]. OSA is an OLA analogue with a methyl side chain instead of the pentyl side chain at C6 of the resorcinolic acid ring, as previously mentioned. In terms of substrate specificity, *Rd*PT1 was characterized to be highly specific for OSA as its prenyl acceptor, while being highly promiscuous for its prenyl donor, using GPP, FPP and geranylgeranyl pyrophosphate (GGPP), although it generally prefers its native FPP substrate for catalysis. Prenyltransferases such as *Rd*PT1 that are able to use longer chain lengths than GPP, such as the C15 FPP and C20 GGPP, are interesting targets of consideration for the diversification of cannabinoid products in the cannabinoid biosynthetic pathway. The longer prenyl chains of CBGA analogues will allow for more complex cyclization patterns in the following oxidative cyclization step catalyzed by cannabinoid synthases. This will, in turn, generate a new class of novel cannabinoids with C15 or C20 substructures at the C3 position of the OLA resorcinolic ring, which may have potential bioactivities that are vastly distinct from the existing class of phytocannabinoids.

Unfortunately, plant aromatic prenyltransferases, such as the aforementioned *Rd*PT1, are usually membrane-associated proteins with typically 7–9 transmembrane helices. The difficulty in elucidating high-resolution 3D structures of these membrane-associated enzymes currently limits mechanistic insight into the structure–activity relationship of these prenyltransferases. Not much is known, except that these plant aromatic prenyltransferases possess two conserved aspartate-rich motifs (e.g., NDxxDxxxD) in protein loops 2 and 6 to coordinate divalent cations, which can stabilize and orientate the pyrophosphate group of the donor substrate [54]. On the other hand, some bacterial and fungal prenyltransferases [58,59,60] that are soluble, well-characterized and with solved structures, may present a better engineering avenue for the prenyl transfer step in cannabinoid biosynthesis.

### 3.2. Alternative Soluble Prenyltransferases

A popular solution for the membrane-associated nature of prenyltransferases from plants is to replace them with a soluble prenyltransferase from bacterial or fungal species. NphB is a well-characterized and soluble prenyltransferase from *Streptomyces* sp. strain CL190 [58]. Kuzuyama et al. characterized NphB to catalyze the transfer of the C10 prenyl group from GPP onto 1, 6-dihydroxynaphthalene (1, 6-DHN) via an Mg^2+^-dependent mechanism and solved the crystal structure of the *holo* enzyme complexed with geranyl S-thiolodiphosphate (GSPP) and 1, 6-DHN. The presence of a large pocket at the aromatic substrate binding site may explain why NphB was substrate-promiscuous for its prenyl acceptors, as it was found to be able to catalyze similar prenyltransferase reactions with a number of different aromatic substrates, including OLA [58]. This gave rise to several reports of different research groups using NphB as a suitable replacement for the prenyltransferase step in the cannabinoid biosynthetic pathway [20,52,61]. However, when catalyzing the reaction between OLA and GPP, NphB was found to produce two products: CBGA, and a side product, 2-*O*-geranyl-olivetolic acid (2-*O*-GOA) [52]. This led to two separate accounts of NphB protein engineering in identifying mutants of the enzyme that has a higher selectivity in producing CBGA [61,62]. Notably, Valliere et al. managed to obtain mutants of NphB (Y288A/G286S and Y288V/A232S) that only produces exclusively CBGA with a 1000-fold improvement in catalytic efficiency. They successfully went on to incorporate their improved prenyltransferase in a cell-free system that produced 1.25 g/L of CBGA from glucose [61]. Further in-depth structure-based studies into the catalytic mechanism of a promiscuous enzyme such as NphB will benefit future efforts in engineering new substrate combinations into the enzyme.

The crystal structure of NphB showed that it exhibits a novel structure consisting of ten strands of anti-parallel β-sheets arranged in a cylindrical manner that was named a PT-barrel. This PT-barrel fold is formed by five repetitive ααββ structural elements and remained exclusive to the ABBA family [63] until the discovery of the dimethylallyl-tryptophan synthase (DMATS) family of indole prenyltransferases, involved in the biosynthesis of pharmaceutically important ergot alkaloids in several fungi belonging to the phylum Ascomycota [64,65]. The ten β-strands arranging in an antiparallel manner forms a central β-barrel enclosing the active site in its spacious lumen, and the α-helices form a solvent-exposed ring surrounding the barrel. Members of these two families exist only in bacteria and fungi, and all of them seem to catalyze the prenylation of aromatic substrates to generate secondary metabolites [66]. They do not contain (N/D)DxxD motifs, and some prenyltransferases such as CloQ are still active in the absence of Mg^2+^ or other divalent cations [59]. These soluble aromatic prenyltransferases show remarkable promiscuity for their aromatic substrates and may be used to biosynthesize libraries of new prenylated aromatic analogues for use in drug development.

**Figure 6:**
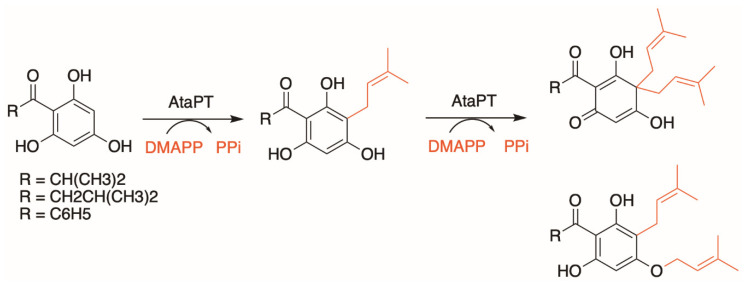
*At*aPT catalyzes predominantly C-monoprenylation of acylphloroglucinols but can also form *gem*-diprenylated products with DMAPP as the prenyl donor, as well as C- and O-diprenylated derivatives.

Another interesting target of study is the aromatic prenyltransferase from *Aspergillus terreus*, named *At*aPT, as it was found to be highly promiscuous for both its prenyl donor (C5–C20) and aromatic acceptors. *At*aPT was reported to be able to catalyze the prenylations of at least 72 aromatic compounds with DMAPP, GPP and FPP [67]. Additionally, apart from the usual single prenylation event reported thus far, where one unit of aromatic substrate is prenylated with one unit of prenyl group, *At*aPT was found to be able to catalyze multiple prenylation events on a single aromatic substrate molecule (Figure 6) [68]. Such an unprecedented promiscuity profile is fascinating as it represents exciting opportunities as a replacement in many biosynthetic pathways that requires a prenyltransferase step, allowing users to produce a vast array of different chemical structures. A comparison of the aromatic acceptor-binding pocket of *At*aPT with those of other DMATS-type prenyltransferases, such as AnaPT from *Neosartorya fischeri* [69] and FgaPT2 from *Aspergillus fumigatus* [60], showed that *At*aPT has an exceptionally spacious hydrophobic substrate-binding pocket, formed by multiple hydrophobic residues with relatively shorter side chains (Gly106, Cys175, Ser192, Gly251, and Gly326) compared to the other DMATS-type prenyltransferases [67]. This provides more flexibility to accommodate various prenyl donors and acceptors, and the hydrophobic nature of the pocket may aid to enhance the stability of prenyl carbocation intermediates, thus facilitating prenylation at multiple sites. Mutagenesis studies of Gly326 in *At*aPT to methionine, to mimic the corresponding Met364 residue in FgaPT2, resulted in a mutant that could no longer accept GPP or FPP as prenyl donors when (+)-butyrolactone II was used as the aromatic acceptor [67]. This demonstrates the possibility of engineering its substrate specificity via the manipulation of active site residues by site-directed mutagenesis.

**Figure 7:**
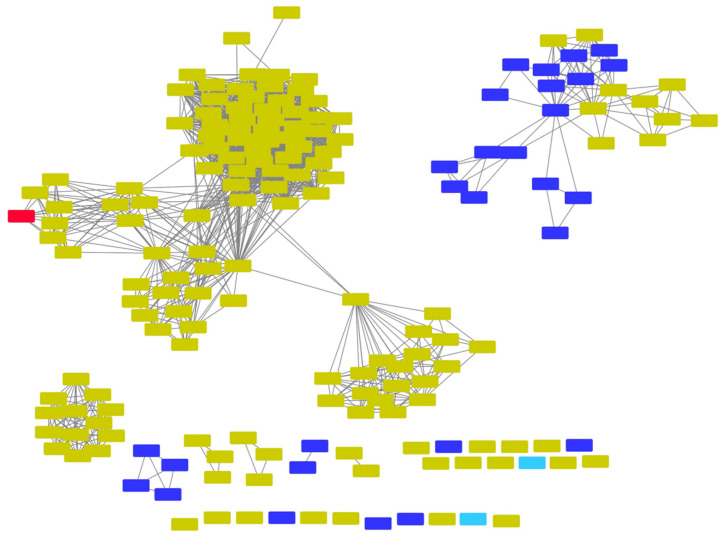
SSN showing putative NphB orthologs belonging to IPR036239 with at least 40% sequence identity to each other. Red: NphB (Q4R2T2) sequence; yellow for bacterial species, dark purple for other *Eukaryota* species, light blue for uncharacterized sequences.

**Figure 8:**
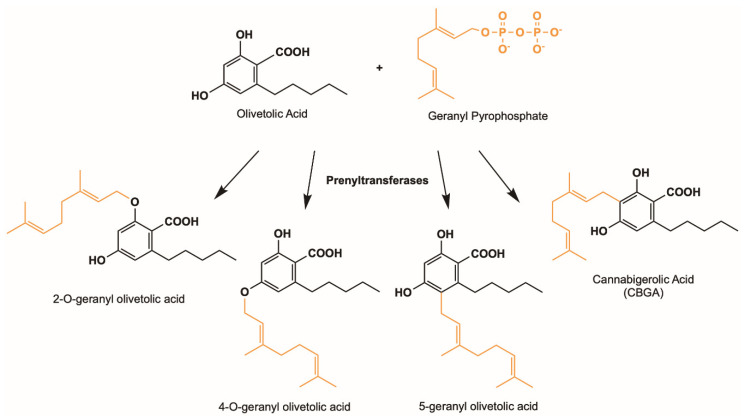
Possible cannabinoid products using OLA (black) and GPP (orange) as substrates.

**Figure 9:**
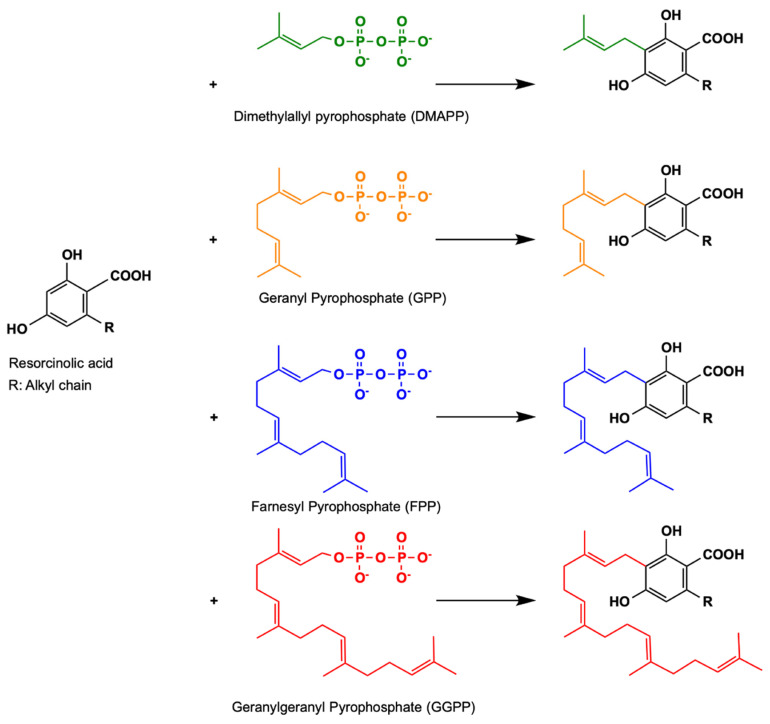
Potential cannabinoid products biosynthesized using an OLA resorcinolic acid scaffold as prenyl acceptor with different prenyl donors.

Soluble and promiscuous aromatic prenyltransferases, such as NphB and *At*aPT, represent only a few of the many sequences found in nature that may catalyze the prenyltransferase step in the cannabinoid biosynthetic pathway. Other uncharacterized prenyltransferase sequences from various sources can be screened for novel functions in relation to cannabinoid biosynthesis. Novel cannabinoid derivatives with different structures can be produced enzymatically; these derivatizations may alter or complement the bioactivity of existing cannabinoid therapeutics. The EFI-EST webtool provides users with an easy way of identifying these related sequences and their relation to each other through the creation of an SSN. Using NphB as a reference sequence, we generated an SSN of related sequences with at least 40% sequence identity to each other in the UniProt database. This allowed us to identify potential orthologous sequences in the database that may perform similar functions, and hence serve as enzymatic replacements in the pathway, or produce novel compounds either from the same substrates or a different set of substrates. Figure 7 shows an SSN generated using the NphB query sequence, identifying 176 sequences, most of which are from bacterial and fungal species. Ongoing studies in our lab have identified several orthologues that are able to catalyze similar reactions using OLA and GPP as substrates to produce CBGA. Additionally, some of these orthologues were found to produce novel products using the same set of substrates, suggesting the ability to prenylate OLA at different positions to give novel CBGA analogues such as 4-*O*-geranyl olivetolic acid and 5-geranyl olivetolic acid, as depicted in Figure 8 (unpublished data). Further studies are underway to uncover prenyltransferase orthologues that are able to catalyze the prenylation using various isoprenoid groups such as DMAPP, FPP and GGPP to an OLA resorcinolic acid scaffold at the C3 position, as depicted in Figure 9. Novel CBGA analogues with either a different side chain on C6 of the OLA resorcinolic acid scaffold, or longer prenyl groups on the C3 position, provide unique cannabinoid scaffolds for testing with downstream cannabinoid synthases in the biosynthetic route.

## 4. Cannabinoid Synthases

The enzymatic reaction catalyzed by the cannabinoid synthases is probably one of the most challenging steps in the cannabinoid biosynthetic pathway. These synthase enzymes, such as the more well-studied THCA synthase and CBDA synthase, are FAD (flavin adenine dinucleotide)-dependent oxidoreductases that are not easy to express heterologously in bacterial systems. The need for post-translational modifications, such as glycosylation and vacuolar compartmentalization for functional expression and protein folding, meant that eukaryotic hosts are arguably the better option [19]. Out of the three cannabinoid synthases identified (THCA synthase, CBDA synthase, CBCA synthase), THCA synthase is the most well-studied as it is the only cannabinoid synthase from *C. sativa* that is crystallized [70] and solved structurally [45]. This is followed by CBDA synthase, which has been isolated, purified and heterologously expressed in yeast and insect cells [27,71]. CBCA synthase has been extracted and purified from young leaves of *C. sativa* and partially characterized in 1998 [72,73]. However, no further in-depth research or reports of heterologous expression has been conducted since.

**Figure 10:**
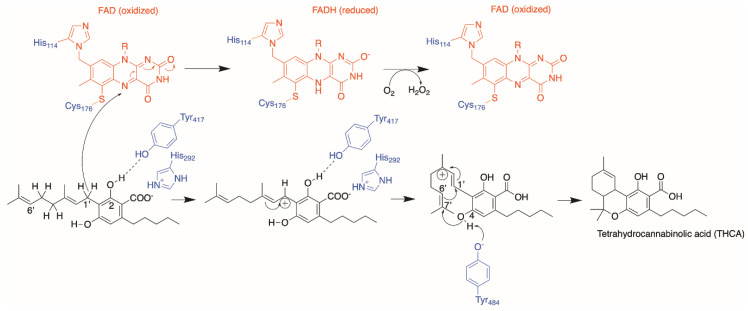
Proposed reaction mechanism of THCA synthase enzyme. The substrate and product are colored black; amino acid residues from THCA synthase are colored blue and the FAD moiety is colored red.

In 2012, Shoyama et al. solved the crystal structure of THCA synthase, allowing us to understand the reaction mechanism from a structural perspective [45]. The oxidative cyclization of CBGA to form THCA was presumed to proceed via two parts: a chemical bond between C1′ and C6′ of the geranyl moiety of CBGA via elimination of the hydride on C1′, and a second chemical bond between the O atom of the hydroxyl group on C4 of CBGA and C7′ on the geranyl moiety through a proton extraction from the hydroxyl group. The FAD moiety was ascertained to be absolutely critical for catalysis and responsible for the hydride transfer on C1′ via the N5 atom of the isoalloxazine ring in FAD, analogous to the reaction mechanism in homologues from berberine bridge enzymes (BBE). Through structural alignments with BBE homologues and further mutational analysis, they concluded Tyr484 is the basic residue in THCA synthase responsible for the remaining deprotonation of the O atom on C4 of CBGA and proposed a reaction mechanism, as depicted in Figure 10.

The high level of sequence similarity (~83%) between the THCA synthase and CBDA synthase indicates that they share a common ancestor [27]. More specifically, it was recently suggested that THCA synthase evolved from CBDA synthase through a duplication and divergence event, giving rise to a different product profile producing dominantly THCA instead of CBDA [74]. Using partially purified protein samples of THCA and CBDA synthases, Zirpel et al. studied their respective product profiles when using CBGA as substrate and found that both enzymes produced the same products, albeit at different ratios, in a pH-dependent manner [71]. They subsequently tried to investigate how differences in amino acid residues in the active sites of both enzymes translate to differences in the product profile. First, an attempt was made to reconstruct a CBDA-dominant product profile using a THCA synthase scaffold by mutating the different amino acid residues in the THCA synthase active site to that of CBDA synthase. Unfortunately, this abolished all catalytic activity which led them to further pursue single amino acid changes of THCA synthase residues to that of CBDA synthase, and vice versa, in a bid to identify the residues responsible for the different cyclization mechanisms. However, despite such an extensive single amino acid search, only the A414V mutation on CBDA synthase slightly altered its product ratio, by increasing the amount of CBDA being produced and shifting its optimal pH from 4.5 to 5.0; the major product that was produced was unchanged. They postulated that differences in product profiles exhibited by the two enzymes might be governed by a key set of residues instead of single amino acid residues in the active site. Another explanation offered was that residues further away from the active site, which were excluded in the experimental design, could play a role in product specificity.

A recently well-studied avenue of derivatization entails the pentyl side chain on C6 of the aromatic resorcinolic ring. This functional group, particularly its alkyl length, has been shown to contribute significantly to the biological effect exhibited by ∆^9^-THC, as demonstrated in the structure–function study conducted by Bow and Rimoldi [75]. This led to various discoveries of phytocannabinoids with different chain lengths; orcinoids with one carbon [76], varinoids with three carbons such as tetrahydrocannabivarin (THCV) and cannabidivarin (CBDV) [77], butols with four carbons [78], hexyl with six carbons [79] and phorols with seven carbon atoms [80]. Citti et al. also confirmed the significance of the biological effect of the side chain when they reported that the analogue of ∆^9^-THC with a seven-carbon side chain, (−)-trans-∆^9^-tetrahydrocannabiphorol (∆^9^-THCP), has a higher CB1 agonist activity in vitro and in vivo [80]. Luo et al. also attempted the heterologous biosynthesis of such analogues at the C6 side chain with varying degrees of chain length, saturation and branching, by feeding their engineered yeast strain with the corresponding precursor fatty acids. Through the successful incorporation of alkene and alkyne groups at the C6 side chain, they further synthetically modified the addition of azide groups through click chemistry [20]. Further discussion will focus on other efforts or possible avenues of diversification through cannabinoid synthases.

### 4.1. Protein Engineering

**Figure 11:**
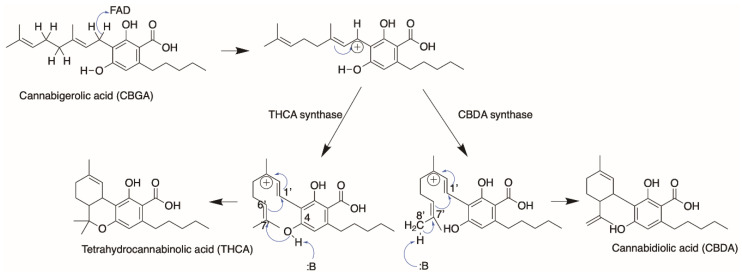
Postulated reaction mechanism between THCA and CBDA synthase forming THCA and CBDA products.

Cannabinoid synthases such as THCA and CBDA synthases are interesting targets of engineering, as these enzymes use the same substrate (CBGA) but produce different products, depending on how they cyclize the C10 geranyl moiety in CBGA. As mentioned prior, the THCA synthase reaction mechanism starts with Tyr484 extracting the proton on O6′, which initiates the reaction cascade that leads to the formation of two chemical bonds. To form a CBDA product in comparison, a chemical bond is formed between C1′ and C6′ of the geranyl moiety in CBGA and an electron transfer from C8 to C9. CBDA synthase is postulated to obtain the proton from C8 of the geranyl group, which subsequently initiates the reaction cascade leading to a CBDA product (Figure 11).

A sequence alignment between THCA synthase and CBDA synthase, without the plastid targeting signal in the first 28 residues, shows that there are 85 amino acid differences. Of these 85 amino acid residue differences (~16.4%), 44 amino acids (~8.5%) are amino acid substitutions with strongly similar properties, 17 amino acids (~3.3%) are substitutions with weakly similar properties, while 24 residues (~4.6%) are non-conserved. A rational protein engineering project aiming to investigate the reaction mechanism pertaining to product specificity of these cannabinoid synthases would be to look at these amino acid differences, starting from the non-conserved residues to the most conserved residues. However, the matrix might still be too large for any efficient screen. Zirpel et al. attempted to narrow down the sequence space by using a targeted approach of looking at amino acid differences close to the THCA synthase active site, but did not manage to find any particular residue or set of residues that are key to this differential product specificity [71].

**Figure 12:**
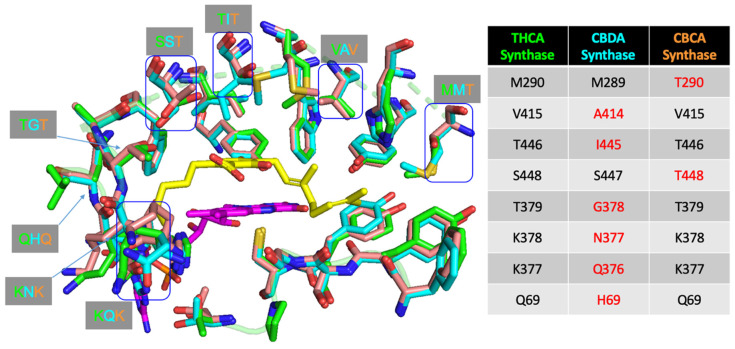
Structural alignment of THCA synthase crystal structure with homology models of CBDA and CBCA synthase showing amino acid differences in the active site. THCA synthase active site amino acid residues are shown in green, CBDA synthase residues shown in cyan and CBCA synthase residues shown in orange. CBGA ligand (shown in yellow) was docked into the THCA synthase active site while the FAD molecule is colored purple.

A closer inspection of the difference in reaction mechanism between THCA and CBDA synthase in Figure 11 reveals the following: (1) both reactions proceed through an FAD-assisted hydride transfer at C1′ of the geranyl moiety on CBGA and an additional base-facilitated dehydration; (2) the two reactions diverge at the position of the proton extraction by the basic residue (proposed to be Tyr484 in THCA synthase and Tyr483 in CBDA synthase, respectively); (3) in CBDA synthase, Tyr483 extracts the proton from the terminal methyl group of the geranyl moiety [27], instead of the O atom of the hydroxyl group on C4 of CBGA in THCA synthase. Taken together, the residues involved in the reaction mechanism are highly similar. However, the architecture of the active site in CBDA synthase, surrounding the geranyl moiety of CBGA, would possibly be different, causing the C10 carbon chain to orientate itself differently, and hence, resulting in the extraction of proton from the end of the chain, instead of the O atom of the C4 hydroxyl group, as seen in THCA synthase. Using THCA synthase as the template, we created homology models of CBDA synthase and CBCA synthase and compared the structures using a structural alignment (Figure 12).

Figure 12 highlights the different amino acid residues found in the active site of the three cannabinoid synthases, analogous to the work of Zirpel et al. However, instead of single amino acid changes, a subset of amino acids could be investigated, such as Val415 and Thr446, changing to the corresponding Ala414 and Ile445, respectively, in CBDA synthase, as these residues are found on the lateral side closer to the prenyl group on the docked CBGA substrate. A sequential step with an increasing number of residues changed could follow until a set of key residues responsible for causing the prenyl group to orientate in a different manner, leading to the difference in cyclization, is found.

Interestingly, Shoyama et al. also investigated the role of the carboxylate functional group in CBGA in catalysis. It was hypothesized that the carboxylate functional group plays a role in substrate binding, through charge complementarity with NH^+^ of the side chain in His292 [45]. This ionic interaction, along with hydrogen bonding with Tyr417, was deemed vital to hold the CBGA substrate in place for catalysis, as mutations on both His292 and Tyr417 reduced the catalytic activity of the enzyme. This could also represent a potential engineering route in designing cannabinoid synthase mutants that are able to accept the decarboxylated CBG as a substrate.

### 4.2. Orthologues of Interest

**Figure 13:**
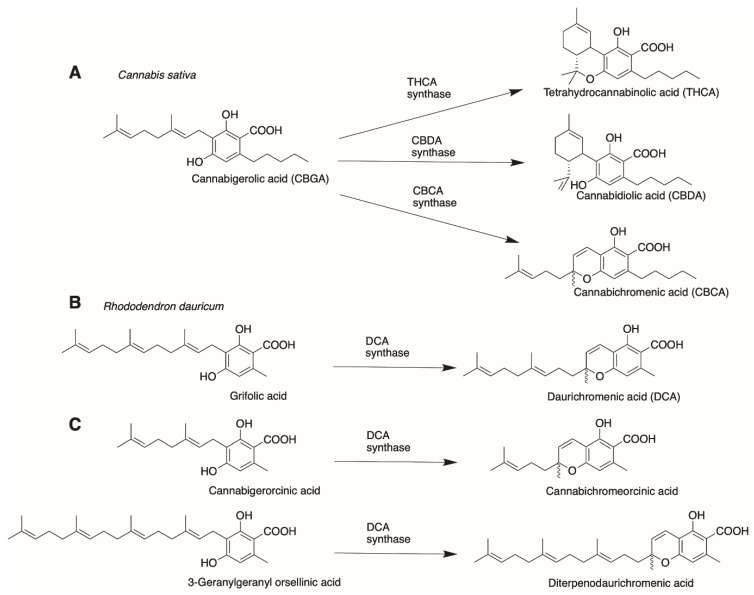
Comparison of naturally occurring substrates and products of (**A**) cannabinoid synthases in *C. sativa* and (**B**) DCA synthase in *R. dauricum* and (**C**) novel DCA synthase with substrates of different prenyl chain lengths.

Lastly, orthologues that evolved from different species can also be tested for bioactivity using CBGA and/or its analogues mentioned earlier as substrates. Gülck and Møller provided an extensive review on phytocannabinoids found in different plant species [5]. However, we wanted to focus on the family of alkylresorcinolic acids produced by *Rhododendron dauricum* as previously mentioned. Like cannabinoid synthases, *R. dauricum* also produces a flavoprotein oxidase that produces daurichromenic acid (DCA) from grifolic acid [81]. Daurichromenic acid chemical synthesis has been extensively studied in recent years [82,83,84] due to its value as a highly effective anti-HIV natural product [76]. In terms of substrate and products formed, DCA synthase is remarkably similar to the cannabinoid synthases. More specifically, the reaction catalyzed by DCA synthase is analogous to CBCA synthase in *C. sativa* producing cannabichromenic acid (Figure 13A,B).

DCA synthase, however, was unable to use CBGA as a substrate. This is possibly due to the pentyl chain on C6 of the resorcinolic ring, as it was able to catalyze cannabigerorcinic acid, an analogue of CBGA with an acetyl group in place of the pentyl chain, to form the synthetic cannabinoid cannabichromeorcinic acid [85]. A further test on substrate promiscuity could be carried out on DCA synthase, using a library of different CBGA analogues with different chain lengths on C6 of the resorcinolic ring, to probe the limits of the enzyme’s promiscuity profile. Interestingly, DCA synthase was observed to exhibit relaxed substrate specificity for substrate analogues with varied prenyl chain lengths, catalyzing the formation of cannabichromeorcinic acid and diterpenodaurichromenic acid, from cannabigerorcinic acid and 3-geranylgeranyl orsellinic acid as substrates, respectively (Figure 13C) [81]. This could represent a potential protein engineering opportunity for DCA synthase, analogous to the examples shown for the prenyltransferases, in engineering the enzyme to be able to catalyze the oxidative cyclization of CBGA analogues with different prenyl chain lengths. CBGA analogues with longer prenyl chain lengths, such as the C15 FPP and C20 GGPP, can be cyclized by orthologous sequences such as DCA synthase, that are able to act on the longer than native C10 geranyl prenyl chain. This potentially represents an interesting route of diversification in the phytocannabinoid chemical space, based on the prenyl chain on C3 of the OLA resorcinolic acid ring, that has not been extensively explored.

**Figure 14:**
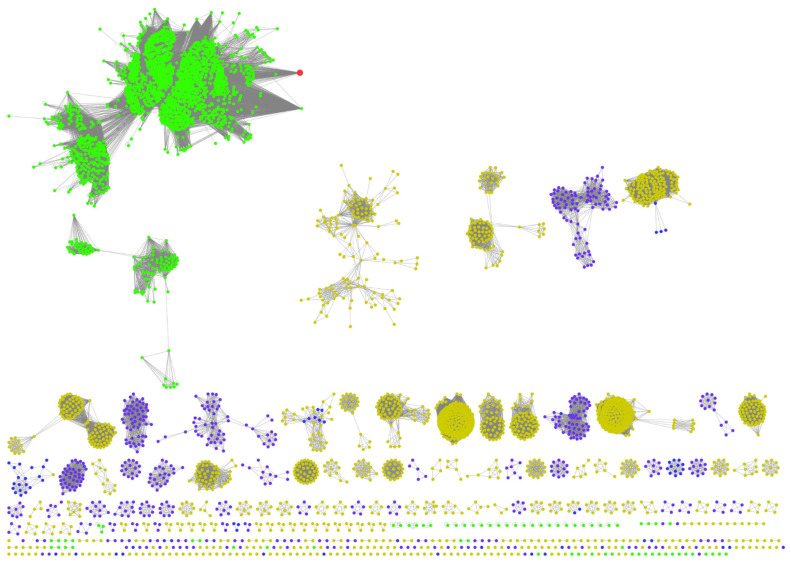
SSN showing putative THCA synthase orthologs with at least 40% sequence identity. Green nodes represent sequences from plant species, yellow from bacterial species, purple from all other *Eukaryota* species. THCA synthase sequence is denoted by a red node.

Cannabinoid synthases belong to a rapidly growing class of flavin oxidases named the vanillyl alcohol oxidase (VAO) protein family [86]. They share a highly conserved FAD-binding domain. Notable members of the group include the berberine bridge enzyme (BBE) from the psychoactive California poppy plant, *Eschscholzia californica*, which catalyzes the formation of (S)-scoulerine alkaloid from (S)-reticuline [87], and carbohydrate oxidases, *Ha*-CHOX and *Ls*-CHOX, from higher plants such as sunflower and lettuce, involved in antimicrobial activity against phytopathogens [88]. Currently identified THCA synthase, CBDA synthase, CBCA synthase and DCA synthase are oxidoreductases from two different species. An SSN generated by using THCA synthase as the query, with 40% sequence identity through the EFI-EST webtool [46], shown in Figure 14, depicts the vast number of other possible orthologues from various natural sources. Each of these orthologues could be screened as a possible replacement in a heterologously assembled cannabinoids pathway, either catalyzing the same reactions in different proportions, or novel reactions giving rise to new products. From the native geranyl moiety of CBGA, there is a limited number of ways in which the C10 carbon chain can be cyclized. However, a longer chain length from farnesyl or geranylgeranyl moieties, such as the ones found in cannabigerorcinic acid and 3-geranylgeranyl orsellinic acid, could expand the repertoire of cyclization paths that a novel synthase identified in the SSN could potentially catalyze.

A large number of these sequences are found in plants, as denoted by the large group of green nodes. This is unsurprising as these secondary metabolites originally isolated from plants were used as a deterrent against herbivory or even as a UV screen that filters UV-B radiation that are biologically harmful [89]. Recently, Go et al. illustrated that possibility by screening a library of 72 orthologues obtained from the EFI-EST webtool using CBGA as the substrate and found six orthologues from various sources that catalyze the formation of cannabielsoic acid (CBEA), a rare cannabinoid found in low abundance in *C. sativa* [90]. This is the first report of using non-canonical flavoproteins outside of the *Cannabis* plant to catalyze the formation of a minor cannabinoid. The emergence of webtools such as the EFI-EST has accelerated the deorphanization of amino acid sequences in databases and will inevitably lead to the discovery of several novel enzymatic functions and products that are not presently reported in nature.

## 5. CB Receptors and Their Ligands

Endocannabinoids and phytocannabinoids are recognized as ligands by several G-protein-coupled receptors (GPCRs) for downstream signaling, to regulate a myriad of physiological processes. As we are concerned about the therapeutic applications of cannabinoids and their novel counterparts, we will limit the discussion to human GPCRs. Understanding how classical phytocannabinoids, such as CBG, THC and CBD, interact and bind to their biological targets in the human body will inspire functional diversity in different functional groups on the phytocannabinoid chemical space.

### 5.1. Classical Cannabinoid Receptors and Their Ligands

Class A GPCRs CB1 and CB2 have been extensively studied in recent years and confirmed as cannabinoid targets. CB2 shares 44% sequence identity with CB1 [91]. CB1 and CB2 have been implicated in many pathological processes, such as pain, epilepsy, anxiety, depression, Parkinson’s and Huntington’s diseases, amyotrophic lateral sclerosis, stroke, cancer, drug dependence, glaucoma, autoimmune uveitis, osteoporosis, sepsis, and hepatic, renal, intestinal and cardiovascular disorders [92]. Although phylogenetically related, they are distributed differently. CB1 is expressed all over the body and to a large extent in the central nervous system (CNS), while CB2 is expressed predominantly in the immune system, and less so in the CNS [93,94,95,96,97]. Due to this difference, it is generally accepted that many side effects of agonist cannabinoid come from CB1 activation [92]; thus there is value in screening a derivatized cannabinoid library to discover cannabinoids with specific binding to CB2 or other candidates, in order to avoid off-target effects.

In terms of structural data, CB1 and CB2 structures have only been resolved in the recent decade. CB1 X-ray crystal structures came earlier (PDB 5TGZ [98], 5XRA, 5XR8 [99], 5U09 [100], 6KQI [101]), followed by those of CB2 (PDBs 5ZTY [102], 6KPC [103]). The cryo-EM structures of both receptors in complex with G proteins were obtained in the past three years: CB1 (PDBs 6N4B [104], 6KPG [103]); CB2 (PDBs 6KPF [103], 6PT0 [105]). GPCRs have seven transmembrane (TM) helices connected by extracellular and intracellular loops. Activation by agonist binding typically involves an agonist binding at an extracellular site formed by gaps between TM bundles, which induces conformational changes facilitating G-protein binding, and leading to signal transduction. Differences in the amino acid sequences of CB1 and CB2 are found in the N-terminal extracellular loop II (ECL2), near the orthosteric ligand binding site, C-terminal of TM7, and C-terminus. Their activation involves key residues termed toggle switches: Phe200 and Trp356 in CB1; Trp258 in CB2. Large relative movements of TM3 and TM6 occur, which opens up the G-protein binding site.

**Figure 15:**
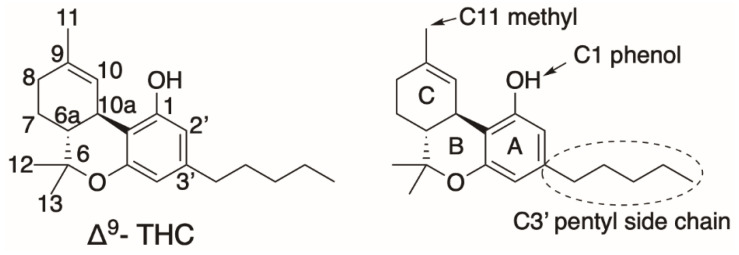
(−)-trans-Δ^9^- THC structural numbering and functional groups.

Known ligands of CB1 and CB2 are generally highly lipophilic in nature. There are indications suggesting that these ligands diffuse laterally from the lipid bilayer, instead of the aqueous extracellular phase [106]. Compared to synthetic cannabinoids, which are full agonists of CB1/2, the classical phytocannabinoid (−)-trans-Δ⁹- THC is a partial agonist of both CB1 and CB2 (K_i_ = 10 nM, 24 nM, respectively [107]). The THC chemical structure consists of a tricyclic core (aromatic A-ring, pyran B-ring, and cyclohexenyl C-ring) and various functional groups (Figure 15). The C3′ pentyl side chain on the A-ring has been the subject of many modifications by different research groups. Increasing its linear length increases THC binding affinity to CB1/2 [75,80], while restricting its flexibility increases affinity when certain conformations are favored. In addition, the substitution of C1′ to bulky ring groups increases affinity and indifference to various heteroatom substitutions, suggesting a CB1 hydrophobic binding subsite near the A-ring, whereas CB2 prefer smaller rings. Removal of the A-ring hydroxyl significantly increases selectivity against CB2. Substitutions of the C-ring C11 methyl group do not affect selectivity between the two receptors but can modulate affinity: Nabilone is a ketone substitution of C11 methyl with high affinity to both CB1/2. The pyran B-ring is not required for CB1/2 activity: CP-55,940, a potent full agonist used as an assay reference, and notably cannabidiol (CBD), has no C-ring pyran core. Bow and Rimoldi provided an excellent comprehensive review regarding various THC analogues and their interactions with receptors [75].

Numerous THC docking and molecular dynamic simulation studies are available for CB1 (as well as CB2, to a lesser extent). Krishna et al. proposed that THC only has CB1 partial agonist activity because of its lack of interaction with the toggle switch [104]. THC also has some conformational flexibility in the orthosteric binding pocket, compared to a highly rigid full agonist. One of THC docking poses in CB1 orients the C3′ pentyl chain at the A-ring towards the toggle switch [104] and hence may explain the higher cannabimimetic activity observed in THC with a longer C3′ side chain [80].

CBD is the second most abundant phytocannabinoid after THC [108]. CBD has recently garnered interest due to its therapeutic potential, especially in epileptic seizures, but its mechanism is still not fully understood [109]. CBD is a partial CB2 agonist [110], and has been reported as an antagonist to CB1 [111], as well as being a CB1 negative allosteric modulator of THC and endocannabinoid 2-AG [112]. A CBD-CB1 docking study identified three putative CBD allosteric sites [113]. Tham et al. proposed that CBD binding at CB1/2 is fluid as CBD seemed to bind at both orthosteric and allosteric sites of CB1, depending on the receptor active state [114]. Chung et al. also showed in MD simulations that CBD and THC can both bind at the same time to the CB1 orthosteric site [115]. For a comprehensive review regarding the structures of CB1/2 and their ligands, refer to the review written by Shahbazi et al. [116].

### 5.2. Orphan GPCRs and Other Cannabinoid-Related GPCRs

Several GPCRs have also been implicated as targets, but none has been IUPHAR designated as cannabinoid receptors, mainly because of data inconsistencies in the pharmacological literature [117]. GPR3, GPR6, and GPR12 share over 60% sequence similarity and are phylogenetically close to CB1 and CB2, belonging to the same MECA cluster (melanocortin, endothelial differentiation, cannabinoid, adenosine GPCRs). They are largely expressed in the brain and the reproductive system and thus, are involved in regulations of various neurological and reproductive processes. GPR3 and GPR6 notably have been reported to recognize CBD [118]. There are several other phylogenetically close GPCRs such as the recently deorphanized free fatty acid receptors and several other orphan receptors [119].

Besides the phylogenetically related GPCRs, more distant receptors such as GPR18 and GPR55 (13% sequence identity to CB1 for both) have also been studied with respect to cannabinoid binding and have been implicated in overlapping pathophysiological processes with CB1 and CB2 [117]. GPR55 is predominantly expressed in the brain and peripheral systems [120,121,122]. It has been shown to bind lysophospholipids [123], while for cannabinoids, some studies report Δ^9^-THC activation [124,125], but functional assays are not consistent [126,127]. GPR18 is highly expressed in lymphoid tissues and moderately in reproductive and several internal organs [128,129]. It has been reported to endogenously bind N-arachidonoyl glycine [129,130] (disputedly, [131]) and Resolvin D2, as well as various cannabinoids [132,133]. None of the abovementioned orphan GPCRs have been resolved structurally, although phylogenetically related GPR3, GPR6, and GPR12 can be modelled with homology modelling tools with reasonable accuracy, due to their relatively high level of sequence identity to solved structures of CB1 and CB2.

CB1 and CB2 themselves may form heterodimers with other GPCRs such as D2 [134,135], CXCR4 [136], or even the abovementioned orphan GPR55 [137], among others [117]. How heterodimerization affects ligand binding directly is not well understood, but oligomerization context has to be considered for therapeutic drug screening efforts. One aminoalkylindole strong cannabinoid agonist WIN55,212-2 has been reported to bind to other GPCRs, dubbed collectively as alkylindole (AI)-sensitive. Likewise, several endocannabinoids, phytocannabinoids, and synthetic cannabinoids have been shown to bind to other GPCRs [138,139,140]. Such cross-talks make neat categorization challenging, but merit consideration.

With diversified libraries of unnatural cannabinoids, it might be desirable to include the less-explored cannabinoid-related GPCRs, or even cannabinoid-binding non-GPCRs, in screening exercises. The emergence of an abundance of unnatural cannabinoid structures with recent metabolic engineering of the cannabinoid biosynthetic pathway may necessitate virtual screening of these unnatural cannabinoids to aid in experimental screening, for instance, by paring down candidates with interaction to these receptors, or to explain mechanisms. Furthermore, investigation of these receptors, if they truly recognize cannabinoids and transduce downstream signals, would shed light on the endocannabinoid system at large, and aid in the discovery and design of novel therapeutic cannabinoids.

## 6. Conclusions

This review summarized the metabolic engineering efforts and the establishment of the phytocannabinoid pathway in heterologous hosts. Understanding of the enzymatic steps in the pathway, along with mechanistic insights, is important in facilitating research in producing novel precursors and cannabinoids through the engineering of substrate selectivity and activity. Orthologous sequences found in nature, that may serve as replacements in the pathway, provide a vast array of opportunities to engineer new substructures with various functional groups into different areas of the phytocannabinoid chemical space. Lastly, we discussed biological cannabinoid targets such as CB1, CB2 and various potential orphan receptors with affinity to cannabinoids, and how classical phytocannabinoids interacted with their receptors in order to inform upstream diversification efforts. Research on cannabinoid biosynthesis has expanded significantly in the recent decade, in response to the recent burgeoning legal *Cannabis* and cannabinoids market. Taken together, this will undoubtedly accelerate our understanding of this unique class of secondary metabolites and their place in modern medicine.
